# Patient Initiated Follow Up for Intellectual Disability Psychiatry Services (Cardiff West): Development of a Quality Improvement Study with Consideration of Patient Reported Experience Measures (PREMs)

**DOI:** 10.1192/bjo.2025.10456

**Published:** 2025-06-20

**Authors:** Lara Wiggins, Catherine Walton

**Affiliations:** Swansea Bay University Health Board, Cardiff, United Kingdom

## Abstract

**Aims:** The rising caseloads in Swansea Bay University Health Board (SBUHB), with some sector consultants managing over 200 patients, exceed the Royal College of Psychiatrists’ recommended caseload of 100. Patients transitioning into Intellectual Disability Services are increasingly complex and therefore need more intensive support which has compounded the issue. This Quality Improvement (QI) project aimed to:

Assess outcomes for patients on Patient Initiated Follow Up (PIFU) within Cardiff West Psychiatry sector of Swansea Bay University Health Board.

Use Patient Reported Experience Measures (PREMs) to evaluate patient and carer satisfaction with the PIFU system.

**Methods:** The Model for Improvement and the Plan-Do-Study-Act (PDSA) cycle were utilised. Planning phase involved stakeholder analysis and process mapping to create a driver diagram identifying problems in the current system. A survey of carers for patients placed on PIFU in 2021–2023 assessed satisfaction with communication and service delivery.

**Results:** In 2021–2022, 9 patients were placed on PIFU; 5 were discharged, and 4 requested a review within 12 months. In 2023, 4 patients were placed on PIFU, with 1 returning for an urgent review. Carer satisfaction was high, with 50% reporting being “very satisfied” and 50% “satisfied”. Regarding communication, 75% of carers felt they understood the PIFU process “very well” and 25% felt they understood it “well”. One carer was unaware that their relative had been placed on PIFU despite generally positive feedback about communication.

**Conclusion:** PIFU has shown potential in reducing caseloads while maintaining high levels of carer satisfaction. The system allowed for appropriate emergency reviews when needed. Some carers expressed a preference for face-to-face follow-up rather than complete discharge. Two barriers were identified. The first, consultant reluctance to discharge due to concerns about vulnerable patients deteriorating without reliable monitoring. The second was administrative staff challenges with IT systems used to track and manage follow-up, in the main due to a lack of awareness of training. Future PDSA cycles will focus on increasing training for both administrative staff and clinicians on the benefits and implementation of PIFU, as well as enhancing carer and patient advocacy.

